# Effect of cold stress on ovarian & uterine microcirculation in rats and the role of endothelin system

**DOI:** 10.1186/s12958-020-00584-1

**Published:** 2020-04-14

**Authors:** Di Wang, Xiumei Cheng, Huimin Fang, Yanqing Ren, Xinhua Li, Weiwei Ren, Bing Xue, Cairui Yang

**Affiliations:** Hebei University of Chinese Medicine, No.326, Xinshi South Road, Qiaoxi District, Shijiazhuang, 050091 Hebei Province China

**Keywords:** Cold stress, Ovary, Uterus, Microcirculation, Endothelin(ET), Endothelin receptor

## Abstract

**Background:**

Cold, an environmental factor, induces many reproductive diseases. It is known that endothelin (ET) is a potent vasoconstrictor, and cold stress can increase the expression of ET and its receptors. The cold stress rat model was developed to examine two parameters: (1) the effects of cold stress on ovarian and uterine morphology, function, and microvascular circulation and (2) possible mechanisms of ET and its receptors involved in cold stress-induced menstruation disorders.

**Methods:**

The rat cold stress model was prepared with an ice water bath. The estrous cycle was observed by methylene blue and hematoxylin and eosin (H&E) staining. Serum estradiol 2 (E_2_), testosterone (T), progesterone (P) were detected by radioimmunoassay. Hemorheology indices were measured. The real-time blood flow of auricle and uterine surfaces was measured. Expressions of CD34 and α-SMA in ovarian and uterine tissues were detected by immunohistochemistry. ET-1 contents in serum were tested, and expressions of ET-receptor types A and B (ET-AR and ET-BR) in ovarian tissues were detected via Western blotting.

**Results:**

Cold stress extended the estrous cycle, thereby causing reproductive hormone disorder, imbalance of local endothelin/nitric oxide expression, and microcirculation disturbance. Cold-stress led to up-regulation of ET-AR expression and protein and down-regulation of ET-BR expression in rats.

**Conclusions:**

This study suggests that the reason for cold stress-induced dysfunction in reproductive organs may be closely related to the imbalance of ET-1 and its receptor expressions, leading to microvascular circulation disorders in local tissues.

## Background

Cold is not only the cause of cardiovascular diseases but also the common cause of many reproductive system diseases. A large number of studies have confirmed that low temperature have adverse effects on the reproductive system. For example, cold exposure can cause an increase in germ cell apoptosis and a reduction in the reproductive capacity of mammals [1, 2]. It also can affect follicular development by activating sympathetic activity in the ovary [3, 4]. In addition, cold not only induces menstrual disorders and dysmenorrhea [5, 6], it can also trigger ovarian insulin resistance, reproductive hormone disorders, and polycystic ovary phenotype [7–9]. Cold exposure can not only induce gynecological diseases via sympathetic neuroendocrine and endocrine systems, oxidative damage, and energy metabolism pathway [10–13], but it can also influence on reproductive system, through blood circulation changes. For instance, research by Meidan et al. showed that cold stress can cause uterine artery contraction, thereby resulting in the reduction of placental blood flow [14]. Friedman et al. found that menstrual cycles are closely associated with the Raynaud’s phenomenon in normal females [15]. Hsu et al. reported that acupuncture of dysmenorrhea rats with condensation syndrome can reduce uterine contractions and increase the uterine microvascular diameters [16]. However, the pathological mechanisms of cold stress-induced reproductive organ blood circulation-induced changes remain unclear.

Endothelin (ET)-1 is a powerful vasoconstrictor peptide secreted by vascular endothelial cells (VECs). It plays an important role in maintaining blood vessel tension by binding with different downstream receptors. When VECs are stimulated, ET secretions are out of balance, vascular tension is abnormal, microvascular lesions, and remodeling occur, all of which result in microvascular circulation disorders [17]. It has been reported that ET-1, a local hormone, can change according to tissues responses to environmental stimuli [18]. Cold exposure not only directly triggers an increase in plasma endothelin secretion but also causes vascular smooth muscle cell contraction, vasospasm, emboli, and even local tissue ischemia and edema. In addition, ET is also expressed in ovarian and uterine tissues [19, 20], which is considered a regulatory reproductive hormone peptide. It plays an important role in regulating reproductive hormone secretion, animal reproductive function, muscle contraction, and cell mitosis. Zhao believes that ET-mediated local vascular regulation disorders and microcirculation disturbances may affect physiological reproductive organ functions [21].

According to the previous experiments, we consider the reason for cold-related menstruation disorder is due to the increase in endothelin content in reproductive organs. Results from previous studies have shown that the content of serum ET increased, nitric oxide content decreased, hemorheology was abnormal, and hemodynamics slowed down in patients with cold-induce irregular menstruation [22, 23]. The increase in ET caused by cold may be closely related to the mechanism of irregular menstruation. What is more, in order to further verify our inference, rat models of cold stress were established in this study. The purpose of this study was two-fold: (1) to explore the effects of cold stress on the ovarian and uterine microvascular circulation in cold-stressed rats and (2) to clarify the role and function of ET-1 and its receptor. Finally, in order to reveal the possible molecular mechanism of cold stress that affects ovarian and uterine functions, the relationship between the cold-related mechanism and endothelin was examined. We speculate that cold stress can cause ovarian and uterine microcirculation disturbances in rats by regulating expressions of ET-1 and its receptor, which will have adverse effects on the reproductive organs of rats.

## Methods

### Major experimental equipment and materials

Neofuge-15R High Speed Refrigeration Centrifuge, Likang Biotechnology Co., Ltd.; DP72 Optical Electron Microscope, Olympus, Japan; DW-86 L386 Ultra low temperature freezer Refrigerator, Qingdao Haier Company; FJ-2021 gamma counter, state-owned 262 plant; LBY-N75008 Automatic Hemorheology Instrument, Beijing Precision Instrument Co., Ltd.; Versa Max Microporous Plate Detection System, Molecular Devices Instruments Co., Ltd.; Moor FLPI-2 Speckle Blood Flow Imaging System, Moore Instruments Co., Ltd.; CV18 Ultrasound Cell Crusher, SONIC, USA; 164–5052 PowerPacTM Universal Power Supply and Trans-Blot@ SD Cell(Bio-rad,USA); ImageQuant LAS-4000 Chemiluminescence Imaging Analyser, GE, Japan.

Lodine [^125^I]-P, E_2_, T Radioimmunoassay (RIA) kit (cat no. PRDRG5, PIDRG6, PIDRG4, Tianjin Jiuding Medical Bioengineering Co.,Ltd.); ET-1 ELISA kit (cat no. S14019020, Cusabio biotech CO.,Ltd.); Universal two-step Kit (cat no. PV-9000, Beijing Zhongshan Jinqiao Biotechnology Co., Ltd); anti-CD34(cat no. SI16–01, Hangzhou Huaan Biotechnology Co.,Ltd.); anti-α-sma (cat no. GB13044, lot no.181407,Servicebio); Goat anti rabbit IgG(H + L)Horseradish Peroxidase(HRP) (cat no.GB23303, lot no.183629, Servicebio), Goat anti Rabblit IgG(H + L) HRP (cat no.GB23303,Servicebio), Goat anti mouse IgG(H + L) HRP (cat no. SA00001–1, proteintech, USA); BCA Protein Quantitative kit (cat no. PQ0011, lot no.82191015, MULTI Sciences), anti-ET-1(cat no.ab117757, lot no.3174228–7, abcam, USA) anti- ET-AR (cat no.ab30536, lot no.222051–9, abcam USA), anti- ET-BR antibody (cat no.ab50658, lot no.3209804–1, abcam, USA); Anti-GAPDH Mouse mAb (cat no.GB12002, lot no.180203, Servicebio); Anti-GAPDH Rabbit mAb (cat no.GB11002, lot no.180203, Servicebio), β-actin (cat no.AC026, lot no.9100026001, AB clonal).

### Animals modeling and grouping

Female Sprague Dawley rats(weighting 180 ± 20 g) in SPF grade were supplied by Beijing Vital River Laboratory Animal Technology (certification number: SCXK-(Jing)2016–0011), and were raised in Hebei Key Laboratory of Liver and Nephropathy Syndrome Research. Rearing conditions is indoor temperature (22 ± 1)°C, humidity (45 ± 5)%, general feed and free drinking water. All experimental procedures were carried out according to protocols, which is in accordance with the National Institutes of Health Guide for the Care and Use of Laboratory Animals. The 32 experimental animals were randomly divided into 4 groups, control group, cold 7d group,cold 14d group,cold 21d group, 8 rats in each group. Cold stimulation was performed on rats, and was carried out by reference [22, 23]. The model groups were swimming in 0–1 °C ice-water at 20 min/d for 7 days, 14 days and 21 days respectively.

### Samples collection and preparation

We collected the blood from the rats during the oestrus. The rats were anesthetized with chloral hydrate(300 mg/kg), monitored local blood flow real-timely, and the blood was collected from the posterior abdominal aorta by vacuum tubes. Part of the blood was put into anticoagulant tubes containing heparin(20 U/ml). Blood rheology was detected and plasma was absorbed (centrifuged,3000 rpm/min 10 min). The serum was obtained by centrifuging another part of the blood. Plasma and serum were stored in − 20 °C refrigerator for radioimmunoassay and ELISA methods. Rats were sacrificed, bilateral ovarian and uterine tissues were dissected, washed with PBS, weighed. One part of the tissue was stored at − 80°Cfor WB detection, and the other part was stored in 4% paraformaldehyde solution for immunohistochemistry. It is necessary to use the smear method of vaginal exfoliated cells to determine the estrous period of rats, once in the period of estrus, we can get materials.

### Estrous cycle, ovarian & uterine index

At the end of 7d, 14d and 21d, vaginal smears were observed every 3 h to determine the estrous cycle. After moistening and disinfecting the cotton swab with normal saline, the cotton swab was rotated and inserted into the vagina of rats, take out the smear after gently rotating in the vagina. When the smear is dry, add 2–3 drops of methylene blue dye, and wash with pure water 15 min later. The types of vaginal exfoliated cells were observed under microscope to determine the estrous stage of rats [24]. Ovarian tissues and uterine tissues were taken and weighed, ovarian index and uterine index were recorded with rat body weight.

### Serum hormones levels

Progesterone(P), Estradiol 2 (E_2_),Testosterone (T) levels in serum were detected with Lodine [^125^I] RIA kit using gammaray radiometer [25, 26]. Sensitivity: P(5 ng/dl), E_2_(2.1 pg/ml), T(1.9 ng/dl). Intra and inter CV: P(10,15%), E2(7.7, 8.9%), T(7.4, 9.8%).

### Histological examination of uterine and ovarian tissues

After dehydration, transparency, wax immersion, embedding, ovaries and uterine tissues were sliced into 4 μm sections and sticked. Consequently, the sections in each group were dyed with hematoxylin, sealed, and observed under microscope. According to the scale (1 unit = 200 μ m), the average diameter (length and diameter) of any 3 follicles and the average height of endometrium of any 3 different parts in each group were measured with Image J software. This is the follicular diameter and endometrial height, respectively.

### Hemorheology index and regional blood flow

The blood samples which were added with heparin were used to detect hemorheological parameters with Automatic Hemorheology instrument. The blood flow of Auricle Microcirculation in rats was monitored in real time and operated strictly according to the instructions of moor FLP. Operate according to the instructions of the instrument. The specific operation methods are as follows: Rats in each group were anesthetized by intraperitoneal injection of 10% chloral hydrate (3 ml/kg). After the rat’s back was fixed on 37 °C constant temperature table, the right ear back was flattened. The CCD camera of the real-time speckle blood flow imaging system is placed 15-20 cm above the auricle, and the focus is clear. Set the parameters of the instrument to high-resolution pixel (752 × 580 pixels) mode, and obtain a blood flow image every second. The acquisition speed is 25 frames/s, the interval time is 1 s, and the exposure time is 20 ms. Each rat was sampled continuously for 10–20s, and three real-time monitoring data with small fluctuation of blood flow were saved.

Real time monitoring of microcirculation blood flow on the uterine surface: Routine disinfection of the abdomen: dissect the abdomen and fully expose the surface of the myometrium to make the uterus as smooth as possible. The blood flow data and blood flow image of uterine microcirculation can be acquired by the same parameter mode, and the blood flow diagram, gray-scale diagram and color diagram of uterine surface microvascular circulation can be obtained at the same time, the color in the Figure shows the instantaneous blood flow, and the size is red > yellow > Green > blue. After the completion of real-time monitoring. The changes of microcirculatory blood flow at different observation points of auricle and uterus were analyzed by Moore flp-2.review v4.0 software. Quantitative analysis of LSI data files. The mean blood flow of each observation point (ROIs) during the monitoring time was calculated.

### Detection of ET-1(ELISA)

ET-1 content in serum and ovarian tissue homogenate were tested with ELISA kit. After strictly following the instructions of the kit, the optical density (OD) of each hole was measured at 450 nm wavelength using the Microplate reader.

### Western blotting analyses

Western blot analysis was performed. First, ovarian tissue proteins were extracted using RIPAlysis buffer containing a protease inhibitor cocktail and PMSF. Proteins were diluted to equal concentration adopting the detection of protein content, using BCA Protein Quantitative kit. The extractive was mixed with 5 × SDS-PAGE sample buffer. Secondly, the samples ware separated by 12% SDS-PAGE and transferred to PVDF membrane, using PowerPac™ Universal Power Supply and Trans-Blot@ SD Cell. After blocking with 5% fat-free milk for 1.5 h, the target protein bands were incubated with antibodies as following, overnight, 4 °C: Anti-ET-1 antibody (1:2000 dilution) Anti-ETA antibody (1:1000 dilution), anti-ETBR antibody (1:1000 dilution) and GAPDH (1:5000 dilution), β-actin (1:20000 dilution). After washing with TBST (5 min, 3 times), the membranes were incubated with secondary antibody(Goat anti rabbit IgG (H + L) Horseradish Peroxidase (HRP), Goat anti mouse IgG(H + L)HRP, for1.5 h at room temperature. At last, after immersing into the ECL luminescence Reagent(Meilunbio, China), the immunoreactive proteins were examined chemiluminescence using Biomolecular imager.

### Immunohistochemistry(IHC)

The immunohistochemical experiment was using the Universal two-step Kit. After dewaxing and rehydration, the ovaries and uterus sections were antigen repaired, then blocked with goat serum (15 min, 37 °C), and Antibody incubation for overnight, 4 °C. Nextly, the sections were incubated with asecondary goat anti-rabbit or mouse IgG(H + L) HRP at 37 °C for 20 min, and stained with DAB for 4–8 s, and counterstained nucleus with heamatoxylin. Subsequently, the sections were covered with coverslips, and observed under optical microscope consequently.

### Statistical analysis

All the data were analyzed by SPSS 19.0. The measurement data were expressed as means ± Standard Deviation, and one-way analysis of variance (*ANOVA*) was used to analyze the difference between groups. Using *P* < 0.05 as the significant difference between the standard.

## Results

### Effects of cold stress on ovarian physiology and function

**Fig. 1:**
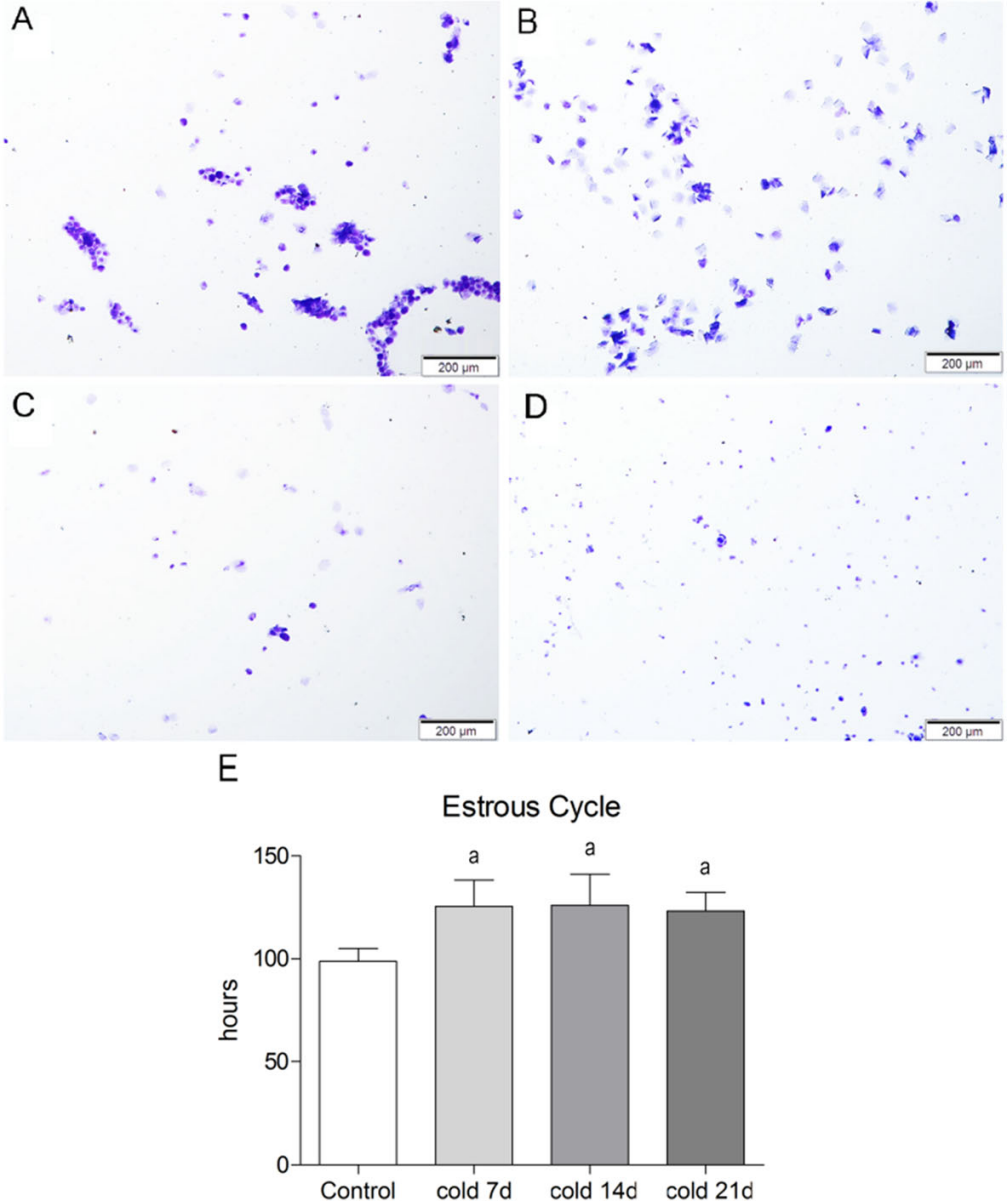
Estrous Cycle and Vaginal Exfoliated Cell Smear in Rats. **a**: Proestrus, **b**: Estrus, **c**: metaoestrus, **d**: anestrum. **e**: estrous cycle(hours), values are means±SD(*n* = 7–8), *p*^a^ < 0.01 vs control

**Fig. 2:**
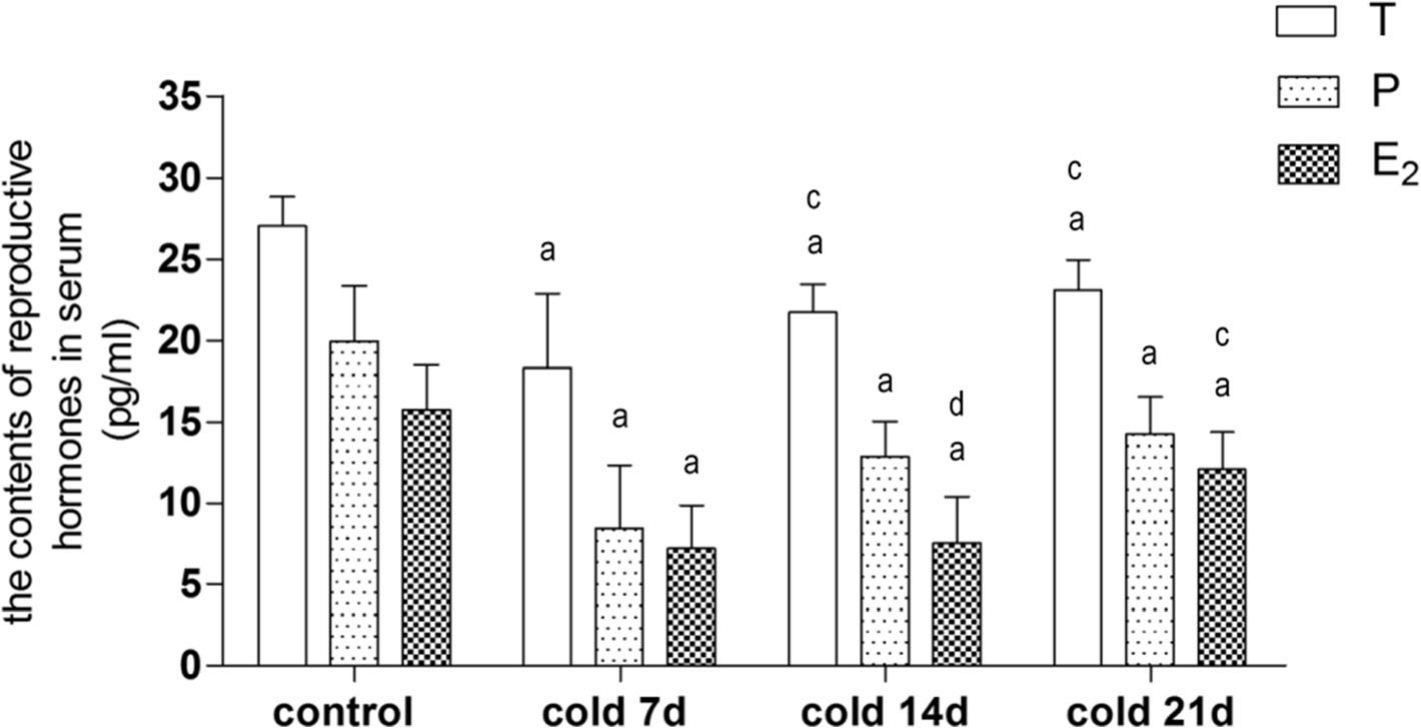
Levels of testosterone(T), estradiol(E_2_) and progesterone(P) in serum of rats, Values are means±SD(*n* = 7–8), *p*^a^ < 0.01 vs control; *p*^c^ < 0.01, *p*^d^ < 0.05 vs cold 7d

The estrous cycle of rats is 4–5 days (96-120 h), which is split into 4 periods: pre-estrous period (Fig. 1a), primarily nuclear epithelial cells; cell estrous period (Fig. 1b); largely nuclear-free keratinocytes; late estrous period (Fig. 1c), white blood cells, keratinocytes, nuclear epithelial cells show no difference. The estrus interval (Fig. 1d) is dominated by a large number of white blood cells. It was found that the estrous cycle of cold-stressed rats was significantly prolonged, with the longest estrous cycle in 7 days group, then shortened slightly after 2–3 weeks of cold stimulation, but still increased significantly compared with the control group (Fig. 1e). Besides, as Fig. 2 shows, the levels of T, E_2_ and P in serum of cold-stressed rats decreased compared with the blank group.

**Fig. 3:**
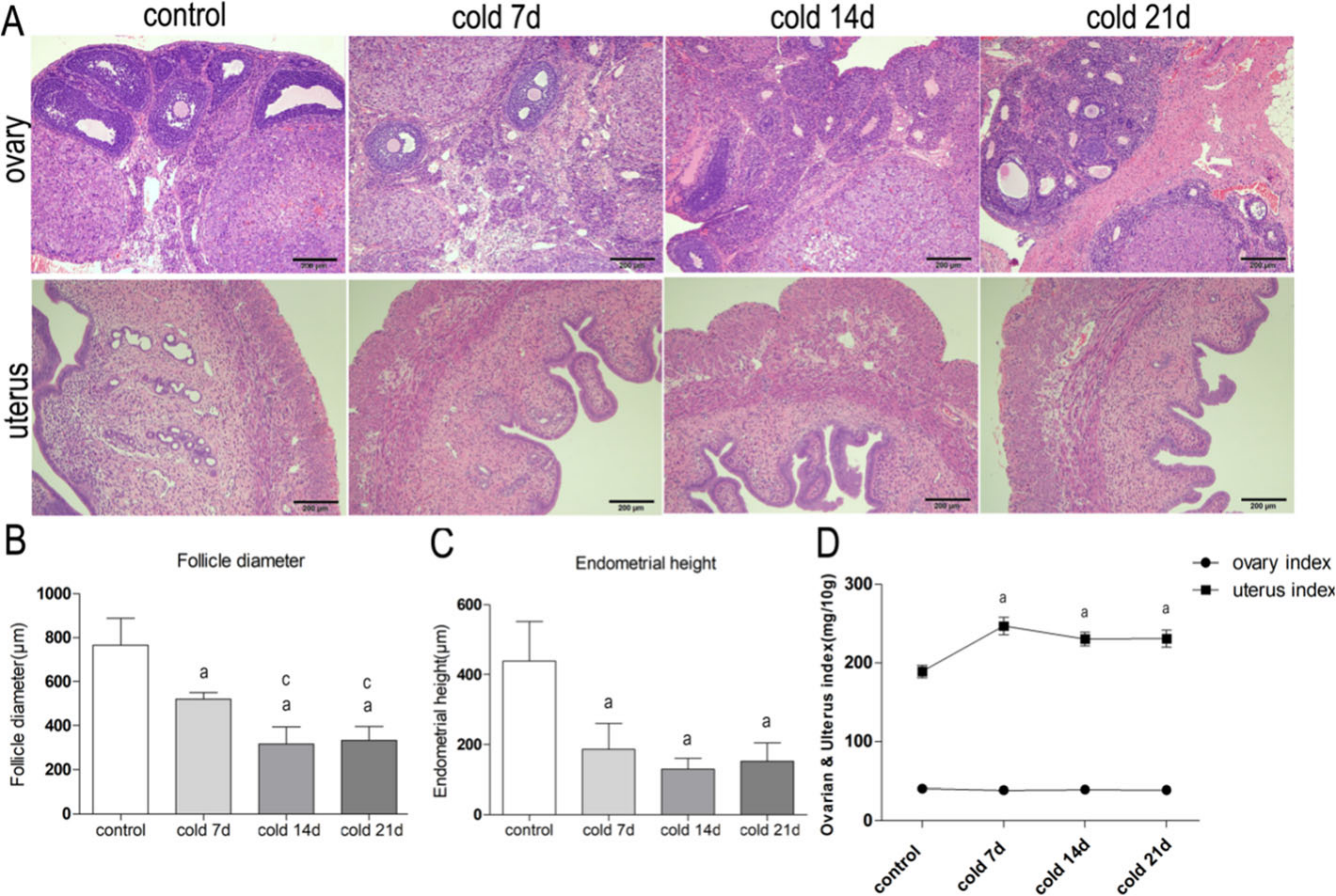
**a**: HE staining of ovary and uterus(100×). Compared with the normal group, the number of primordial follicles and primary follicles in each cold group increased significantly. The diameter of granulosa layer and theca cell layer of ovary decreased obviously. In the cold stimulation groups, the lumen of uterus was narrow, and the lumen epithelium of endometrium was irregular papillary. **b**: Comparison of ovarian follicle diameter in each group, values are means±SD(*n* = 3), *p* < 0.01^a^ vs control, *p* < 0.01^c^ vs cold 7d. **c**: Comparison of endometrial epithelial height in each group, values are means±SD(*n* = 3), *p* < 0.01^a^ vs control. **d**: Comparison of ovarian index and uterine index in each group, values are means±SD(*n* = 3), *p* < 0.01^a^ vs control

Effects of cold stimulation on histopathology of ovary and uterus in rats (Fig. 3): In the cotrol group, the ovarian structure was normal, and there were follicular cells and luteal cells in each stage. The granulosa cells in the follicles surrounded the oocytes and arranged closely, and there were transparent bands and radiating crowns between them. The number of follicles decreased significantly in each cold group. In addition, the diameter of granulosa layer and theca cell layer of ovary was significantly smaller than that of control group, especially in 7d group and 14d group. In the control group, the structure of endometrium was normal, there were high columnar epithelium, stromal cells and glands. There are smooth muscle layer and squamous cell layer on myometrium and serosa of uterus. The lumen of uterus in each cold stress group was narrow, and the epithelium of lumen of endometrium was irregular papillary. The endometrium and myometrium are edematous and the glands are reduced or irregular (Fig. 3a).

Compared with the control group, the follicle diameter of each cold group was significantly smaller than that of the control group (Fig. 3b) (*P* < 0.01). The height of endometrial layer decreased significantly (all *P* < 0.01) (Fig. 3c). There was no significant difference in ovarian index between the groups. It may be related to the small base of ovary weight. However, the index of uterus in cold stimulation group increased significantly (Fig. 3d) (*P* < 0.01).

### Effect of cold stress on blood flow in rats

**Fig. 4:**
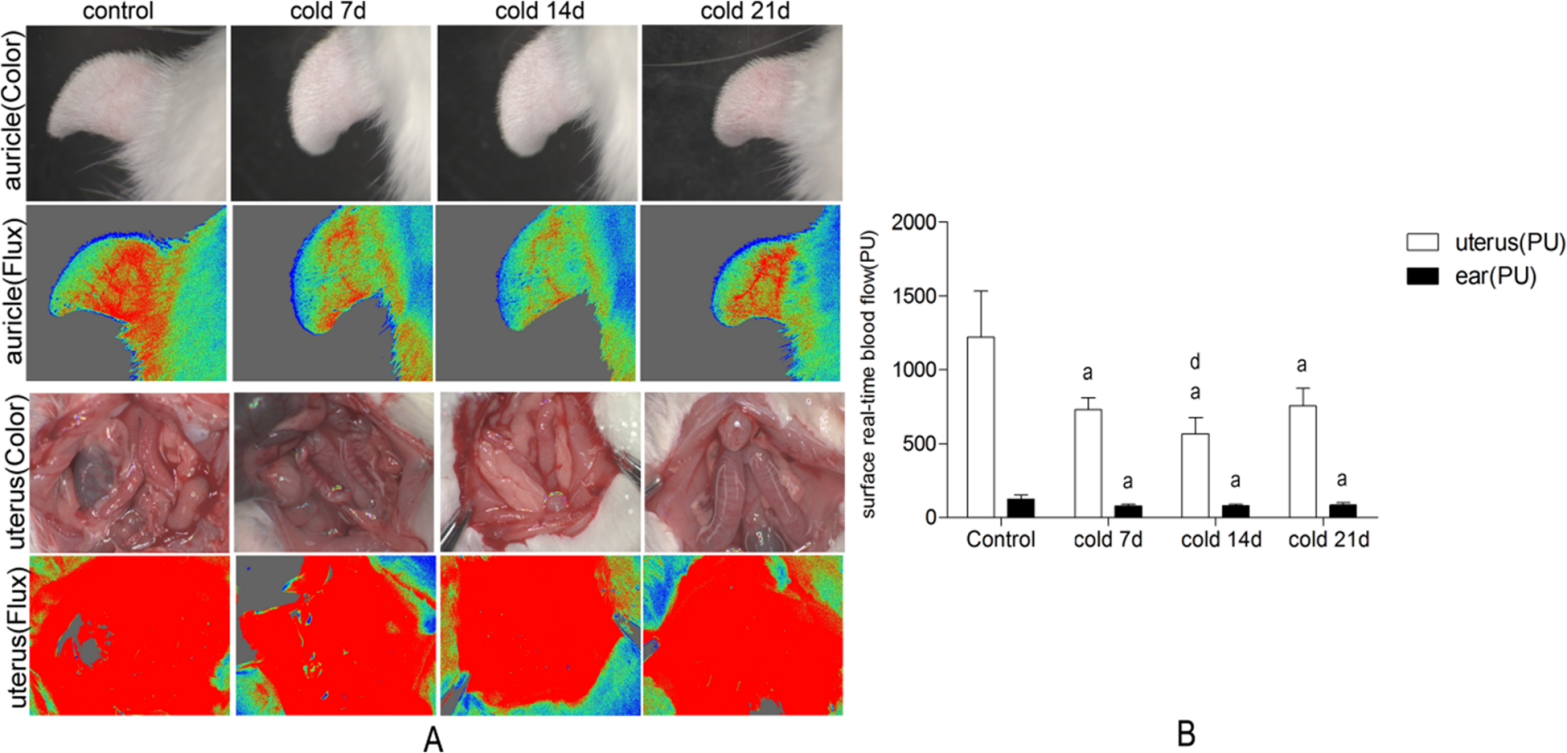
Laser speckle real-time imaging in groups. Cold stress reduces auricular microcirculation and uterine surface microcirculation blood flow in rats. **a**: surface Flux&color figures for auricle and uterus in each group. **b**: real-time blood flow (PU). Values are means±SD(*n* = 7–8), *p*^a^ < 0.01 vs control, *p*^d^ < 0.05 vs cold 7d

Compared with the control group, the hemorrheology had changed after 7 days of cold stress. The whole blood low shear, high shear, whole blood reduced viscosity and erythrocyte aggregation index were significantly increased in the 14 days of cold stimulation group (Table 1). There was no significant difference between the other cold stimulation groups and the blank group. At the same time, the microcirculation of auricle and uterus surface of rats in each cold stressed group was monitored in real time. Blood flow measurements were significantly slowed down (Fig. 4).

**Table 1:** Hemorheology indexes in each group, values are means ± SE (*n* = 7–8), *p*^a^ < 0.01, *p*^b^ < 0.05 vs control

Group	Whole blood			Blood reduced viscosity			Erythrocyte aggregation
	High(150)	Middle(60)	Low(100)	High	Middle	Low	
Control	3.63 ± 0.54	5.13 ± 0.33	10.67 ± 0.97	7.13 ± 2.29	9.98 ± 2.48	20.38 ± 2.51	2.65 ± 0.30
Cold 7d	4.18 ± 0.90	5.81 ± 0.12^*^	12.39 ± 2.11	8.25 ± 0.51	11.13 ± 0.65	22.74 ± 1.46	3.07 ± 0.73
Cold 14d	4.91 ± 0.44^*a*^	6.84 ± 1.12^*a*^	15.95 ± 3.60^*b*^	9.82 ± 0.88^*b*^	13.69 ± 2.25^*a*^	31.90 ± 3.20^*b*^	3.52 ± 0.54^*b*^
Cold 21d	4.38 ± 0.63	5.73 ± 1.02	12.62 ± 1.35	8.75 ± 0.27	11.45 ± 2.04	25.58 ± 2.18	2.76 ± 0.15

### Immunohistochemical analysis of ovarian CD34 and α-SMA

**Fig. 5:**
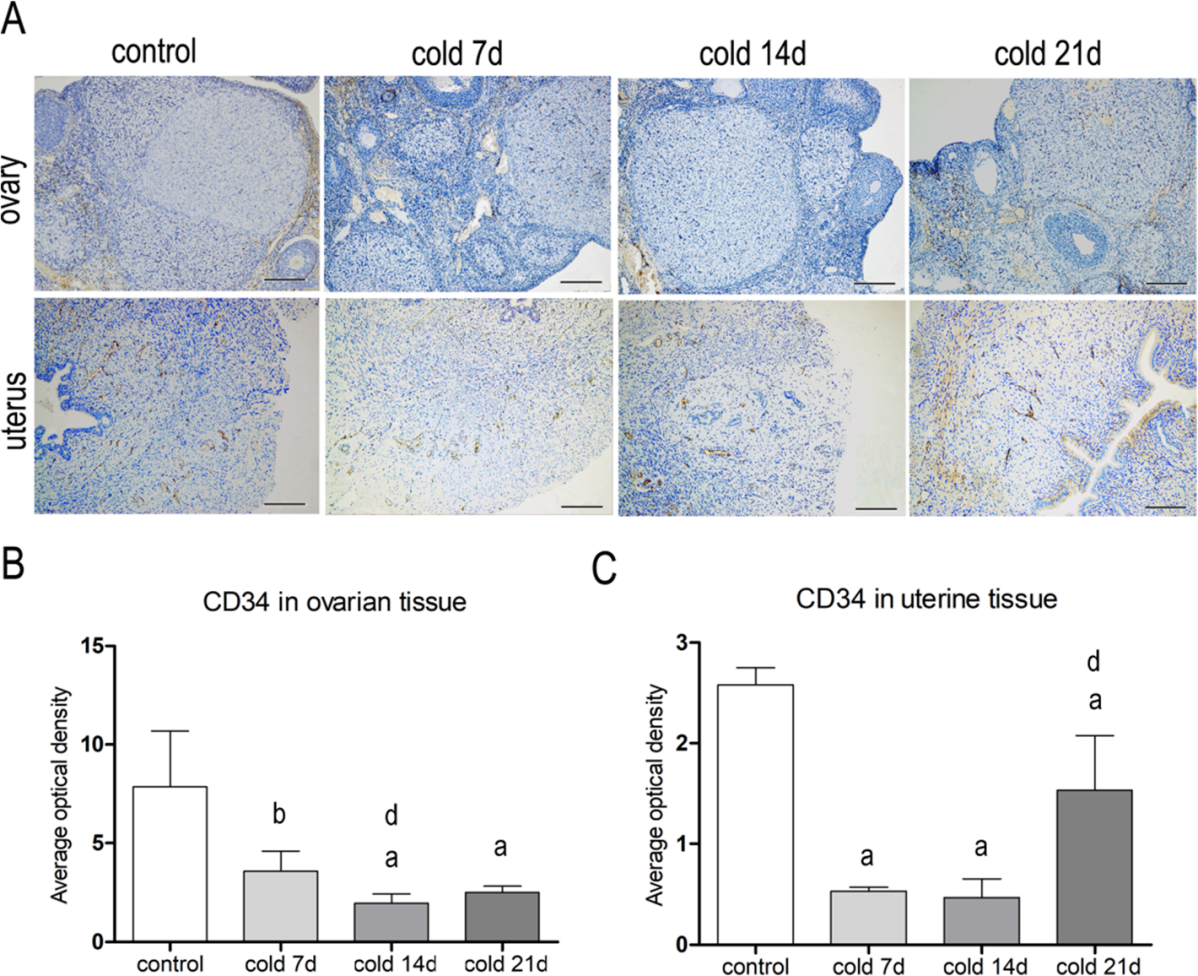
**a**: The positive expression of CD34 antibody in ovarian and uterine tissue(× 100). and the positive area was in the brown area, mainly expressed in the cytoplasmIn and the membrane of vascular endothelial cells. **b**: Relative optical density value of anti-CD34 in ovarian tissue; **c**: Relative optical density value of anti-CD34 in uterine tissue, values are means±SD(*n* = 3),*p* < 0.01^a^, *p* < 0.05^b^ vs control; *p* < 0.05^d^ vs cold 7d

**Fig. 6:**
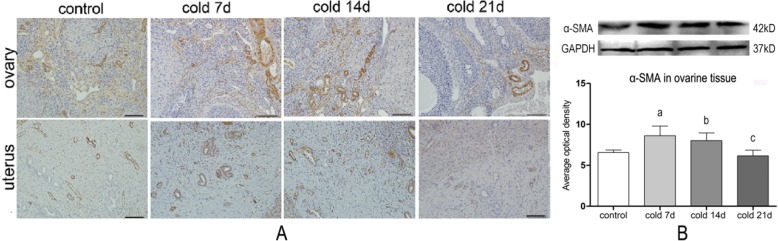
**a**: The protein localization of α-SMA in ovarine and uterian was detected by IHC(200×), and the protein positive area was in the brown area, mainly expressed in the cytoplasm of vascular smooth muscle cells. **b**: Relative optical density value of α - SMA protein in ovarian tissue was detected by WB, values are means±SD(*n* = 3) *p* < 0.01^a^, *p* < 0.05^b^ vs control, *p* < 0.01^c^ vs cold 7d

CD34 and α-SMA were expressed in vascular endothelial cells, in uterus and vascular smooth muscle cells of rats’ ovary and uterus (Figs. 5 and 6), respectively. Compared with control group, the positive rate of CD34 in ovary and uterus for cold groups was decreased (*P* < 0.05), and the expressions of a-SMA protein in ovary of the cold groups were increased (*P* < 0.05). It is suggested that cold stress can down-regulate the CD34 positive rate and up-regulate the expression of a-SMA protein in ovary and uterus.

### Effect of cold stress on ET-1 expression in serum and ovarian tissue of rats

**Fig. 7:**
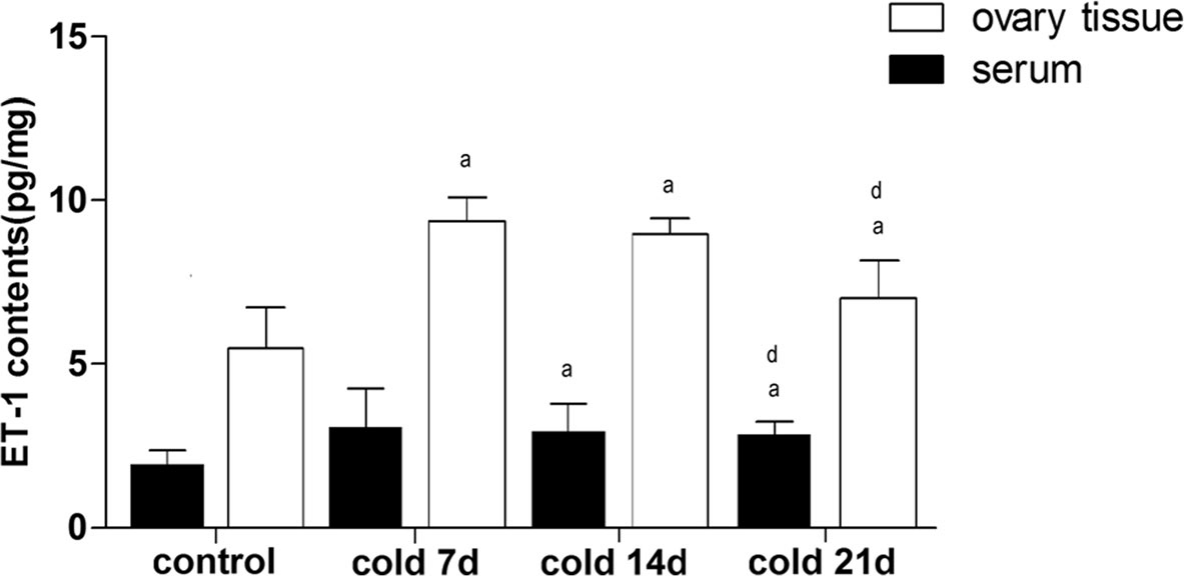
ET-1 contents in serum and ovarian tissue of rats. Values are means±SD(*n* = 7–8), *p*^a^ < 0.01, *p*^b^ < 0.05 vs control, *p*^d^ < 0.05 vs cold 7d

There was no significant difference in the content of serum ET-1 among the groups, indicating that cold had no significant effect on the expression of serum ET-1. However, the ET-1 content of ovarian tissue in each cold group increased, the highest in cold 7 days, about twice as high as that in control group, and began to decline after the end of cold 14 days (Fig. 7). It is suggested that cold can increase the content of ET-1 in ovarian tissue, which proves that ET-1 is a local hormone rather than a circulating hormone.

### Effect of cold stress on the protein expressions of ET-1 and ET-AR and ET-BR in ovarian tissue

**Fig. 8:**
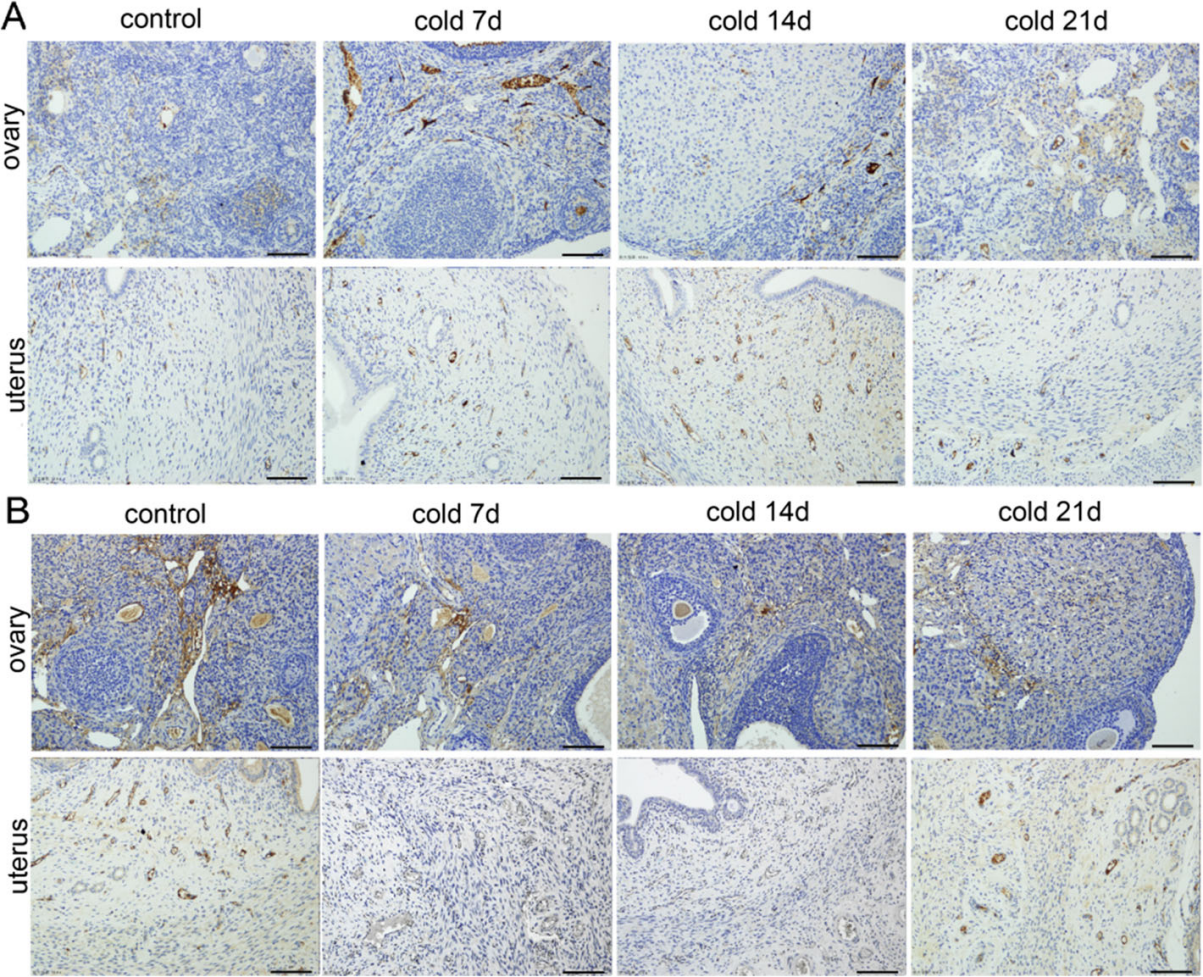
ET-AR and ET-BR proteins in ovary and uterus for each group by IHC(200×). **a**: ET-AR was expressed in ovarian vascular endothelial cells and uterine vascular endothelial cells. **b**: ET-BR was expressed in ovarian oocytes and vascular endothelial cells, uterine epithelial cells and vascular endothelial cells

**Fig. 9:**
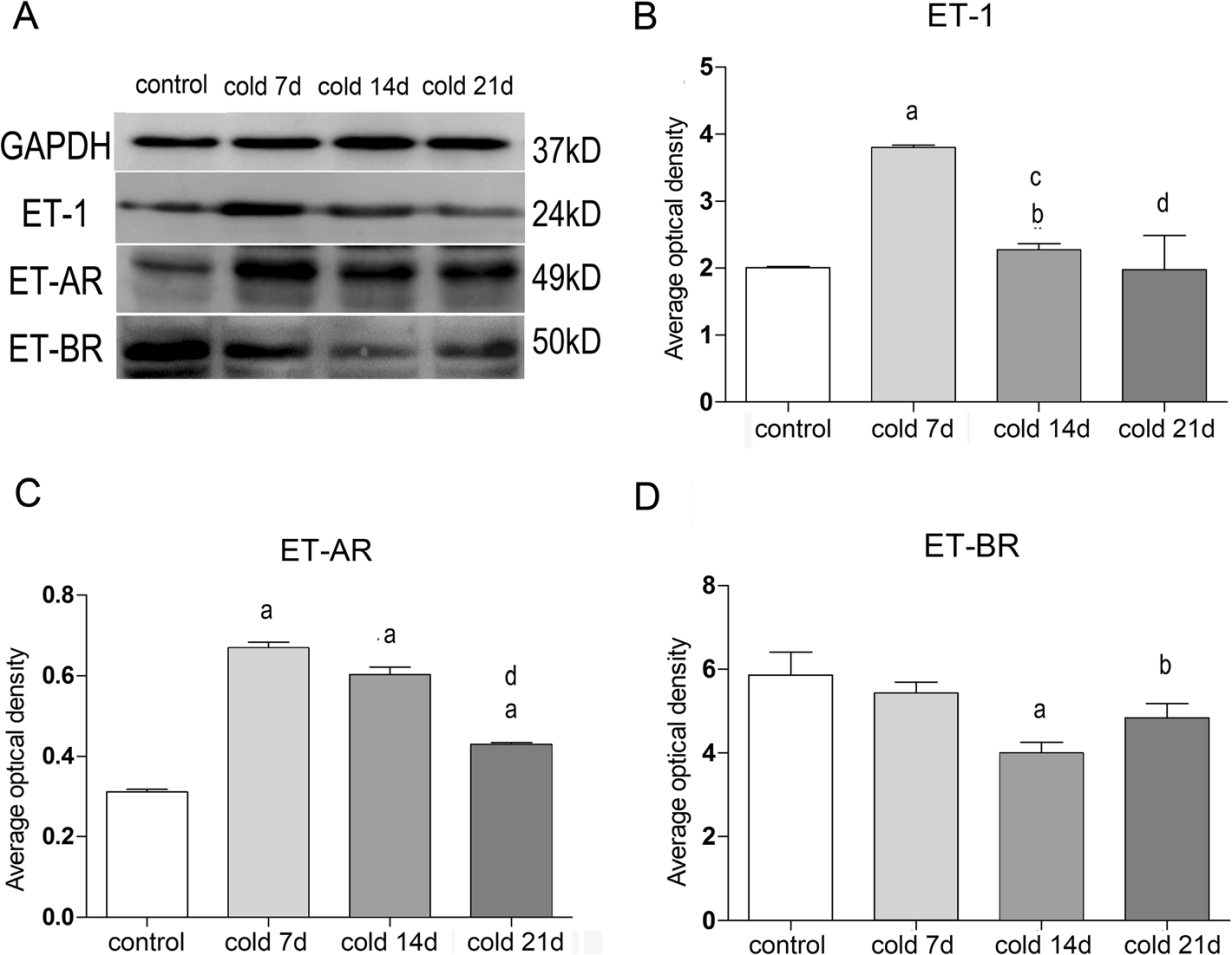
**a**: Gray band of GAPDH, ET-1, ET-AR, ET-BR protein. **b**, **c**, **d**: Relative optical density value of ET-1, ET-AR, ET-BR for each group by Western Blotting analysis, values are means±SE(*n* = 7–8), *p*^a^ < 0.01, *p*^b^ < 0.05 vs control;*p*^c^ < 0.01, *p*^d^ < 0.05 vs cold 7d

As shown in Fig. 8, ET-AR and ET-BR are expressed in ovary and uterus. ET-AR was expressed VECs in ovarian and uterine tissue, ET-BR was expressed oocytes and VECs in ovarian, and epithelial cells and VECs in uterine. Compared with control group, the expression of ET-AR in cold stimulated rats was significantly higher (*P* < 0.05 or *P* < 0.01). However, the expression of ETBR decreased significantly (*P* < 0.05, *P* < 0.01). There was a positive correlation and a negative correlation with the expression level of ET-1 in tissues (Fig. 9). Among them, the difference was the biggest in cold 7d group, and the difference decreased with the increase of cold stress time. It is suggested that cold can up regulate the expression of ET-1 and its a receptor, down regulate the expression of B receptor. According to the results, cold stress could induce abnormal physiological functions of ovary and uterus in rats. It may have a relation to the cold regulation of endothelin system, VEC injury and local microcirculation disturbance of ovary and uterus.

## Discussion

Our experiments showed that in rats stimulated with cold stress, cold stress could not only prolong their estrous cycles and reduce testosterone, estradiol, and progesterone levels. Cold stress could also cause physiological reproductive organ dysfunction, which has adverse effects on hemorheology and microcirculation. On the other hand, cold stress caused a reduction in the percentages of (CD)34-positive cells, up-regulation of the α-SMA protein expression in ovarian and uterine tissue, and up-regulation in the content and expression of ET and ET-AR proteins. Cold stress also promoted ovarian and uterine vasoconstriction and spasms. Therefore, our results show that the abnormal physiological ovarian and uterine function caused by cold stimulation may be related to the local ovarian and uterine microcirculation disorders caused by cold regulation of the ET system.

Cold stress of rats produced with an ice water bath was the method applied in the early experiments by the research group. The method is different from the freezing method, which is a local and mild cold stress induction method. The cold stress model has been cited by many researchers and belongs to the recognized method of cold coagulation stasis syndrome model in traditional Chinese medicine [27–29]. However, adrenaline, instead of subcutaneous injection of epinephrine, is the cause of blood stasis; thus, we used an ice water bath as a direct cold stimulus to induce reproductive stress in rats and examine whether it could affect body circulation and local reproductive organ structure and function [30]. We used ice water bath method to immerse rats in water at 0 °C, the water level was low, about 4 cm. The water level setting is the distance that the water submerges the abdomen when the rat is upright. This method did not cause rats to swim in the water, but made ice water directly stimulate the abdomen, that is, the uterus and ovary of rats.

Our results showed that in the cold group, the estrous cycle was disrupted, ovarian follicle diameter decreased, and the endometrial layer became thinner. It is suggested that cold may have adverse effects on ovarian function and lead to periodic uterine changes. We collected the serum from the rats during the oestrus. At this time, the content of estradiol represents the basic level of the body, cold group is compared with the normal group, the measured estradiol level decreased. This indicated that the basic level of estradiol in cold group decreased, correlated with hypohormonal cycle. The levels of reproductive hormones were measured in rats in the estrous interval. The decrease of estrogen level indicates ovarian dysfunction. The stages of the sterus cycle for the rats in different groups at the time of tissue and blood harvesting is in the period of estrus. The basic levels of serum testosterone (T), progesterone (P), and E2 also decreased in varying degrees, which suggests that cold stress can cause a decrease in ovarian reproductive endocrine function. What is more, low temperatures have bad effects on the reproductive system. For example, cold exposure can induce the polycystic ovary phenotype by activating the sympathetic nervous system and changing the development of ovarian follicle [8, 10, 31] and cause an increase in germ cell apoptosis and decrease mammalian reproductive capacity [1–3]. Reproductive hormone disorders also affect follicular development by regulating and activating ovarian sympathetic activity [5]. The research results of Xu et al. show that the cold exposure-induced uterine morphological changes may be due to the effects of P, which was also confirmed by our study [32].

After 7 days of cold stress, there were no significant hemorheological changes, but the microvasculature blood flow to the auricle and uterine surfaces had begun to decrease. It is suggested that the cold stress may cause local microvascular constriction and stenosis of the peripheral resistance vessels, which has little effect on the circulation vessels. However, changes in ovarian and uterine shape and function occurred on cold day 7. It is suggested that cold stress-induced microvascular disturbances and blood supply decrease to reproductive organs may be the cause of the morphological and functional changes of reproductive organs. In addition, CD34, as a specific marker of VECs, is mainly expressed on the endothelial cell membranes. CD34 is usually used to label micro-vessels and endothelial cells [33, 34]. α-SMA, as a specific marker of vascular smooth muscle cells (VSMCs), indicates VSMC contraction and mesenchymal cell proliferation [35, 36]. The results of this study confirm that the ovarian and uterine CD34-positive cell percentages decreased, and the expression of α-SMA smooth muscle actin increased. Consequently, it is confirmed that cold stress may damage blood vessel endothelial cells, reduce the microvasculature, and increase VSMC proliferation. Ovarian and uterine vascular remodeling, vasoconstriction, and spasms may occur.

It has been reported that there is a close relationship between microvascular circulation and reproductive system [37, 38]. Vascularization is an important part of ovarian morphogenesis. Pascuali et al. believed that the vascular system has a great influence on the formation of ovarian structure, early development process, lifeline formation, primordial follicle collection, and follicular activity [39]. Parborell et al. pointed out that by inhibiting angiopoietin activity, the number of atretic follicles mediated by ovarian apoptosis increased [40]. Cold can also cause a reduction in uterine blood flow and increase in uterine contraction, leading to post -placental hemorrhaging and endometrial morphological changes in rats [32, 41]. In addition, Meirow’s experiment explained that the change in ovarian resistance index in endometriosis is related to interstitial fibrosis and microvascular damage [42]. Therefore, the results of this study showed that cold stress-induced microcirculation disturbances might cause the low reproductive function of cold-stimulated rats.

ET-1 is the most potent vasoconstrictor; it is mainly secreted by vascular endothelial cells and plays an important role in the circulatory system [43–45]. The vasoconstrictors synthesized and released by VECs increased, and at the same time, secretion of vasoactive substances, such as prostacyclin and nitric oxide, decreased, and G-protein-dependent extracellular signal transduction pathway defects increased. The levels of adhesion molecules increased, which leads to an increase in vascular tension and platelet aggregation. At the time, VSMC phenotypic transformation and proliferation, vasoconstriction, and pipe diameter decrease eventually lead to microvascular reconstruction and microcirculation disorders [46–48].

Studies have shown that cold stress can directly cause changes in ET endothelin levels in the mesenteric resistance artery, and it can regulate protein expression of the AB receptor in the renal cortex [18]. Our previous research results demonstrated that the content of serum ET increased, nitric oxide content decreased, and hemorheology changed in patients with cold coagulation and stasis syndrome. Thus, it is suggested that the cold-induced ET increase may be closely related to the pathogenesis of menstrual disorders [22, 23]. However, according to the results, cold stress could up-regulate ET-1 expression in ovarian tissues, yet it had no obvious effect on ET-1 expression in serum. However, it was verified that ET is not a circulating hormone [49]. Thus, the content of ET-1 in ovarian tissue plays a more important role when evaluating the cold-induced ovarian and uterine injury.

ET-1 plays a vasoconstrictor role by increasing the intracellular Ca^2+^ concentration of ET-AR and also plays a vasodilator role mediated by ET-BR [49–51]. Therefore, using immunohistochemistry and Western blotting we confirmed the expression of ET-1 and its receptor protein in ovary and uterus. In addition, we found that the expressions of ET and its receptor protein in ovarian tissue was disrupted by cold stress. Because the activated ET-1 protein binds more to the ET-AR protein, we only detected the location of the receptor protein in tissues. ET-AR was expressed in ovarian and uterine VECs. ET-BR was expressed in oocytes and VECs in ovarian tissues and epithelial cells and VECs in uterine tissues. ET-AR protein increased significantly from day 7 after cold stress, while the ET-BR protein decreased from day 14 after cold stress (this may be related to the increase in total ET-1), which positively correlated with the ET-1 expression level in the tissues and negatively correlated with ET-1expression level. It is suggested that cold stress can cause up-regulation of the expressions of ET-1 and its receptor, down-regulation of ET-BR expression, and cause local microvascular ovarian and uterine spasms and contractions.

It has been confirmed by various researchers that ET-1, as a reproductive hormone regulatory peptide, also regulates the hypothalamus-pituitary-gonad axis (HPG), which is closely related to the physiological ovarian and uterine function and plays an important role in mammalian reproductive processes. There are mature and dense capillary networks in the luteal body. In granulosa cells, ET can promote vasoconstriction, lead to tissue ischemia and hypoxia, suppress P secretion by luteal cells, and cause deterioration of luteal function, thereby affecting follicular development [52]. In addition, when menstruation culminates, ET-1 and other vascular effectors in endometrial stromal cells act together with P and transforming growth factor (TGF)-beta, to regulate blood flow of spiral arterioles, leading to endometrial ischemia, exfoliation, and menstruation [43]. ET-1 expression is different during the menstrual cycle. ET-1 may be involved in periodic changes in the human endometrium, such as proliferation and decidualization. Temporary spaciotemporal expression of two endopeptidases (endothelin converting enzyme [ECE]-1 and neutral endopeptidase [NEP]) involved in the synthesis and degradation of ET-1 may be involved in regulating the role of ET-1 in the human endometrium [53, 54].

Therefore, we believe that cold stress causes activation of the ET-1 protein in ovarian tissues. By increasing the binding of ET-1 to ET-AR and decreasing its binding to ET-BR, ET may cause local microvascular contraction, thinning, vasospasms, and vascular remodeling, thus decreasing blood flow. This ischemic and hypoxic microenvironment will eventually lead to the ovarian and uterine dysfunction, which is manifested in the decrease of follicle diameter, endometrial thickness, reproductive hormone levels, and estrous cycle disorders. In conclusion, the imbalance of ET-1 and its receptor expression caused by cold stimulation may be one of the important mechanisms of the local ovarian and uterine microcirculation disturbances in cold-stressed rats.

Accordingly, this study shows that the abnormal function of ovary and uterus caused by cold stimulation is closely correlated with the local microcirculation disturbance caused by endothelin system in ovarian and uterine tissues. It is noteworthy that this study clarifies the effect and mechanism of cold stimulation on the reproductive system and organs from microcirculation and proposes that improving local microcirculation is vital to maintain the physiological function of ovary and uterine. However, the mechanism of cold-induced endothelin expression in ovaries and uterine and whether there is fibrosis in cold-damaged ovary and uterine need further studies.

## Conclusions

The research shows that cold stress can prolong the estrous cycle and cause the reproductive hormone disorder and low ovarian function in rats. Cold affects ovarian physiological function, which may be associated with endothelin system, in other words, the up-regulation of ET-1 and ET-AR and the down-regulation of ET-BR protein expression. This will mediate the vascular contraction and the microcirculation disorders in ovaries and uterine of rats.

## Data Availability

All data generated or analysed during this study are included in this published article [and its supplementary information files].

## Abbreviations

α-SMA: α-smooth muscle actin
ET-AR: Endothelin A receptor
ET-BR: Endothelin B receptor
VEC: Vascular endothelial cell
VSMC: Vascular smooth muscle cell
P: Progesterone
E_2_: Estradiol 2
T: Testosterone
ELISA: Enzyme-linked immunosorbent assay
PMSF: Phenylmethylsulfonyl Fluorid
PVDF: Polyvinylidene Fluoride
GAPDH: Glycerol 3 phosphate dehydrogenase
